# Time perception of individuals with subthreshold autistic traits: the regulation of interpersonal information associations

**DOI:** 10.1186/s12888-022-03995-z

**Published:** 2022-05-27

**Authors:** Bin Xuan, Shuo Li, Peng Li, Lu Yang

**Affiliations:** 1 School of Educational Science, Anhui Normal University, Wuhu, 241000 China; 2 Institute of Artificial Intelligence, Hefei Comprehensive National Science Center, Hefei, 230088 China

**Keywords:** Autistic traits, Time perception, Temporal bisection task, Identity-association learning

## Abstract

**Background:**

People with high subthreshold autistic traits usually share behavioral patterns similar to those of individuals on the autism spectrum, but with fewer social and cognitive changes. The effect of autistic traits on time perception and the role of interpersonal information in this effect remain unexplored.

**Methods:**

This study used a temporal bisection task between 400 and 1600 ms to compare the time perception of individuals with higher and lower autistic traits, and to explore the regulation of interpersonal information on their time perception by establishing associations between identities and geometric shapes. Thirty-two participants with high autistic traits and thirty-one participants with low autistic traits participated in this study.

**Results:**

In the absence of identity information, people with high autistic traits tended to judge short durations as longer. Their subjective bisection point was lower, and the Weber ratio was higher than for those with low autistic traits, suggesting that their overestimation of short duration was due to decreased temporal sensitivity. With the involvement of interpersonal information, the proportion of long responses for no identity was significantly lower than for self, friends, and strangers, which seemed more obvious in individuals with low autistic traits although there was no significant interaction between identity and group. The Weber ratio of no identity was lower than that for other identities.

**Conclusion:**

The results suggest that individuals with high autistic traits have more conservative responses that are relatively shorter in duration, and this change is related to a decline in perceptual sensitivity. Compared to individuals with high autistic traits, the time perception of individuals with low autistic traits seemed more susceptible to interpersonal information.

## Background

Autistic traits refer to a set of behavioral, personality, and cognitive traits associated with autism spectrum disorders (ASDs) [1], which are widespread and continuously distributed in both individuals with ASD and the general population [2, 3]. People with high levels of autistic traits include both clinical ASD patients and subthreshold individuals with high levels of autistic traits who do not meet the clinical diagnostic criteria. The two groups usually share similar behavioral patterns but show different degrees of changes in social and cognitive functions [4, 5]. Therefore, the studies on subthreshold individuals with high autistic traits are valuable to promote understanding of and intervention for individuals with ASD, even though the concept of “autistic traits” is still controversial [6, 7].

Changes in social function in clinical individuals with high autistic traits are mainly characterized by social and speech disorders [8], which may be closely related to their ability to represent and process interpersonal information and their reduced sensitivity to social information. “Self” and “others” are two typical interpersonal roles, and individuals with high autistic traits show some atypical characteristics in their representation and processing of interpersonal information. Many studies have noted that people with high autistic traits show more extreme self-centeredness [9, 10]; however, it has also been found that individuals with ASD show a decentration trend in self-processing [11–14]. In processing the faces and names of oneself and close ones, individuals with ASD do not show an obvious “self-preference effect” as typically developing individuals [11]. As an extension of research on self-processing, a study on the ownership effect has also yielded interesting results. Grisdale et al. (2014) found that typically developing adults were better at remembering objects belonging to themselves, and that typically developing children rated objects belonging to themselves as more attractive and valuable than those belonging to others [13]. However, children and adults with ASD did not show a similar ownership effect. In addition, typically developing individuals showed co-activation in the right prefrontal cortex when seeing the faces of self and others, whereas fMRI studies have revealed that children with ASD do not show these co-activations, which may be the underlying mechanism of understanding the commonality between self and others [14]. These results suggest that atypical self-processing and other processing occur simultaneously in individuals with high autistic traits. A better understanding of the self often means a better representation of others’ minds and better processing of interpersonal information [15, 16]. It should be noted that the level of autistic traits may play an important and subtle role in the processing of interpersonal information. Gillespie-Smith et al. (2018) suggested that clinical individuals with higher autistic traits tend to show an “absent self,” while clinical individuals with lower autistic traits were less likely to pay attention to others and showed a higher self-bias tendency [17].

Researchers are more likely to emphasize changes in social communication rather than in basic cognitive function for individuals with high autistic traits, such as time perception. Many everyday activities require us to organize our behaviors with respect to time [18], and time processing is also the basis of many advanced cognitive functions, such as attention, memory, emotion, and language. This is crucial for an individual’s cognitive development [19]. However, previous studies have reported conflicting results. Some scholars have found that subthreshold high autistic traits may lead to enhanced time perception. The higher the level of autistic traits, the more likely they were to form a stable and accurate representation of pitch and time to auditory stimuli [20]. It has been reported that the temporal estimation of individuals with ASD is intact in the sub-second range, if not enhanced [21]. However, an emerging body of research suggests that temporal processing may be disrupted in individuals with ASD. Self-reports of individuals with ASD, as well as reports from relatives or physicians who have frequent contact with them, suggest that they have changed their time perception in their daily lives [22]. Using a questionnaire and open-ended questions, Poole et al. (2021) found that behaviors related to time can have a considerable impact on the daily lives of children with autism [18]. Vogel et al. (2019) reported a distinct pattern of interrupted-time experience from qualitative data acquired from 26 adults with high-functioning ASD [23]. Some researchers have suggested that changes in time processing were one of the key characteristics of individuals with ASD [24, 25]. Jurek et al. (2019) and Casassus et al. (2019) reviewed timing-related studies and indicated that there remains no clear consensus on whether or how timing mechanisms may be affected in autism [22, 26]. However, Jurek et al. (2019) also indicated that time processing in individuals with ASD was defective in almost all temporal ranges, thus showing greater variability, higher discrimination thresholds, and lower accuracy [26]. However, previous studies on different time ranges showed that the mechanisms of autistic traits were not the same for short- and long-term processing. Thus, children with high functioning ASD showed relatively significant errors in time replication tasks of both 500 ms and 45 s; however, the errors for short time were related to basic sensory processing and timing pulse release, and the errors for a long time may be related to higher cognitive processing, such as attention and memory [27].

As a breakthrough in the understanding of cognitive processing mechanisms in clinical ASDs, Nijhof and Bird (2019) found that autistic traits affect not only the processing of social information but also the broader, varied domains of cognitive processing [28]. Time perception is a fundamental cognitive ability, and previous studies on time perception related to autistic traits have also concentrated on individuals with high autistic traits. In the current study, we focused on whether and how interpersonal information can modulate the relationship between subthreshold autistic traits and time perception. More broadly, it is important to explore whether and how the influence of autistic traits on the processing of social information further affects basic cognitive processes. Because temporal processing may cover a relatively wide range, we limited temporal processing to the perceptual range by using a temporal bisection task in the 400-1600 ms range. In addition, we specifically introduced geometric shapes as visual stimuli that matched physical attributes and established their associations with the self and others. Based on the results of previous studies, we hypothesize that compared to individuals with low autistic traits, non-clinical individuals with high autistic traits may show decreased sensitivity in time perception. Individuals with low autistic traits are more susceptible to identity information when interpersonal information is involved. Instead, because individuals with high autistic traits are not sensitive to interpersonal information, they may show relative stability in time perception.

## Experiment 1

### Participants

A total of 280 revised Chinese versions of the Autism Spectrum Quotient (AQ) questionnaires were distributed among undergraduates and postgraduates using convenience sampling, and 249 valid questionnaires were obtained. The groups with high level and low level of autistic traits (hereinafter, “high-AQ group” and “low-AQ group”) were based on grouping criteria from previous studies [29–31]. According to previous studies [32–34], taking the proportion of excluded participants in the group with high autistic traits into account, the total sample size was calculated by G*Power 3.1.9.4, when α = 0.05, power = 0.80, and selected approximately 30 participants for each group. Therefore, based on total scores, the top and bottom 27% of the individuals were selected, combined with the AQ score range, and according to the willingness of the participants, 63 participants were finally recruited to participate in the study. There were 32 participants (9 males and 23 females) in the high-AQ group with an average age of 20.65 ± 1.51 years and average AQ scores ranging from 125 to 161, with an average score of 130.03 ± 5.94. In the low AQ group, there were 31 participants (14 males and 17 females) with an average age of 19.39 ± 1.66 years and an average AQ score of 102.87 ± 4.48, in the range of 89–110. An independent samples *t*-test showed that the AQ scores of the two groups were significantly different. All participants had normal or corrected visual acuity, were right-handed, and volunteered to participate in the experiment. After the completion of the experiment, the participants were able to obtain the corresponding compensation. All participants signed an informed consent form before the experiment, and the study met ethical standards.

### Methods

#### Measures

The autistic spectrum quotient (AQ) is a widely used tool for measuring autistic traits. It was developed by Baron-Cohen et al. (2001) and is mainly used to measure the level of autistic traits in non-clinical populations [2]. Austin (2005) first proposed a Likert scoring method for the Autism Spectrum Quotient, where individual items are scored from 1 to 4 [35]. The revised Chinese version by Zhang et al. (2016) was used in this study [36]. AQ includes the following five dimensions: social skills, attention to conversion, attention to detail, communication, and imagination, with a total of 50 questions. Each question had four options, from “completely agree” to “completely disagree,” using a 4-point Likert-type scale. The total score ranged from 50 to 200; the higher the score, the higher the level of autistic traits. The internal consistency coefficient of the revised Chinese version was 0.81, the retest reliability was 0.89, and the internal consistency coefficient of each subscale was between 0.62 and 0.76. The internal consistency coefficient of the AQ in this study was 0.71.

#### Procedures

**Fig. 1:**
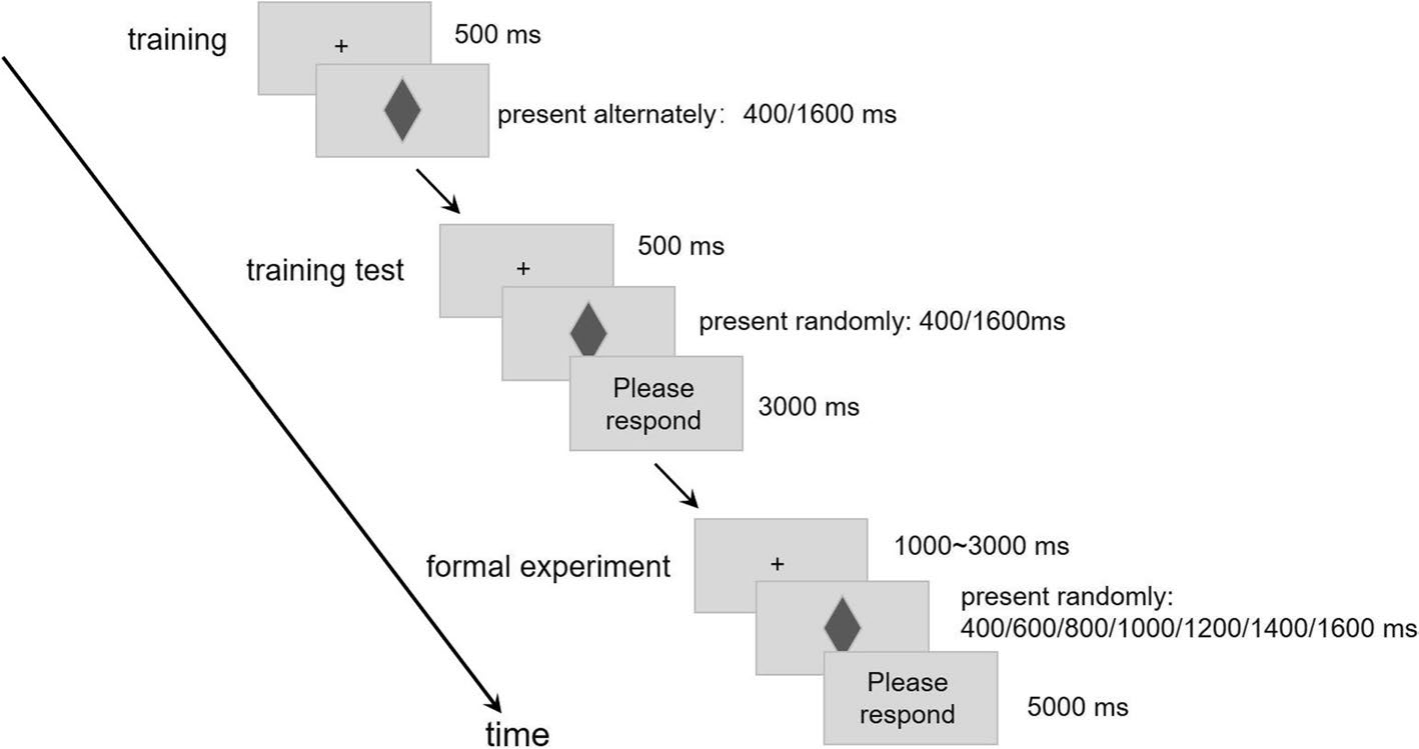
Procedure of Experiment 1

Experiment 1 used a temporal bisection task [37], which was divided into three stages: training, training test, and formal experiment. During the training stage, gray diamonds (with a long diagonal of 5 cm and a short diagonal of 3 cm) were alternately displayed in the center of the screen at 400 ms and 1600 ms, five times each. The participants were informed that 400 ms was the standard short duration, and 1600 ms was the standard long duration. At this stage, participants felt and tried to remember the standard long and short durations, but no response was required. In the training test stage, gray diamonds with standard long or short durations were randomly presented in the center of the screen, and the participants were asked to judge “long” or “short” duration length, pressing the “F” or “J” keys, respectively, to respond. Feedback was provided based on the participants’ responses at this stage. After 10 training trials, the participant could only enter the next stage if all choices were correct. In the formal experimental stage, the visual stimulus appeared at the center of the screen, randomly, in one of the seven intervals of 400, 600, 800, 1000, 1200, 1400, and 1600 ms, and the participants were asked to judge whether the time interval was closer to the standard long duration or short duration. There were 196 trials in the formal experiment; each duration was randomly presented 28 times, and the interval between trials was random within 1–3 s. Thus, the procedure for Experiment 1 is illustrated in Fig. 1.

#### Data statistics

The proportion of long responses, response time, subjective bisection point, and the Weber ratio were calculated according to the judgments made by the participants. According to Droit-Volet et al. (2004), the proportion of long responses refers to the proportion of the frequency whose duration is judged to be close to a long duration to the total frequency under a certain condition [37]. The subjective bisection point was the duration corresponding to the proportion of a long responses of 0.5. The Weber ratio is defined as half of the difference between the durations corresponding to the proportions of long responses of 75 and 25%, divided by the subjective bisection point. In this study, the sigmoid function was used to fit the proportion of long responses, and the subjective bisection point and Weber ratio of each participant were calculated using the fitting function.

### Results

**Fig. 2:**
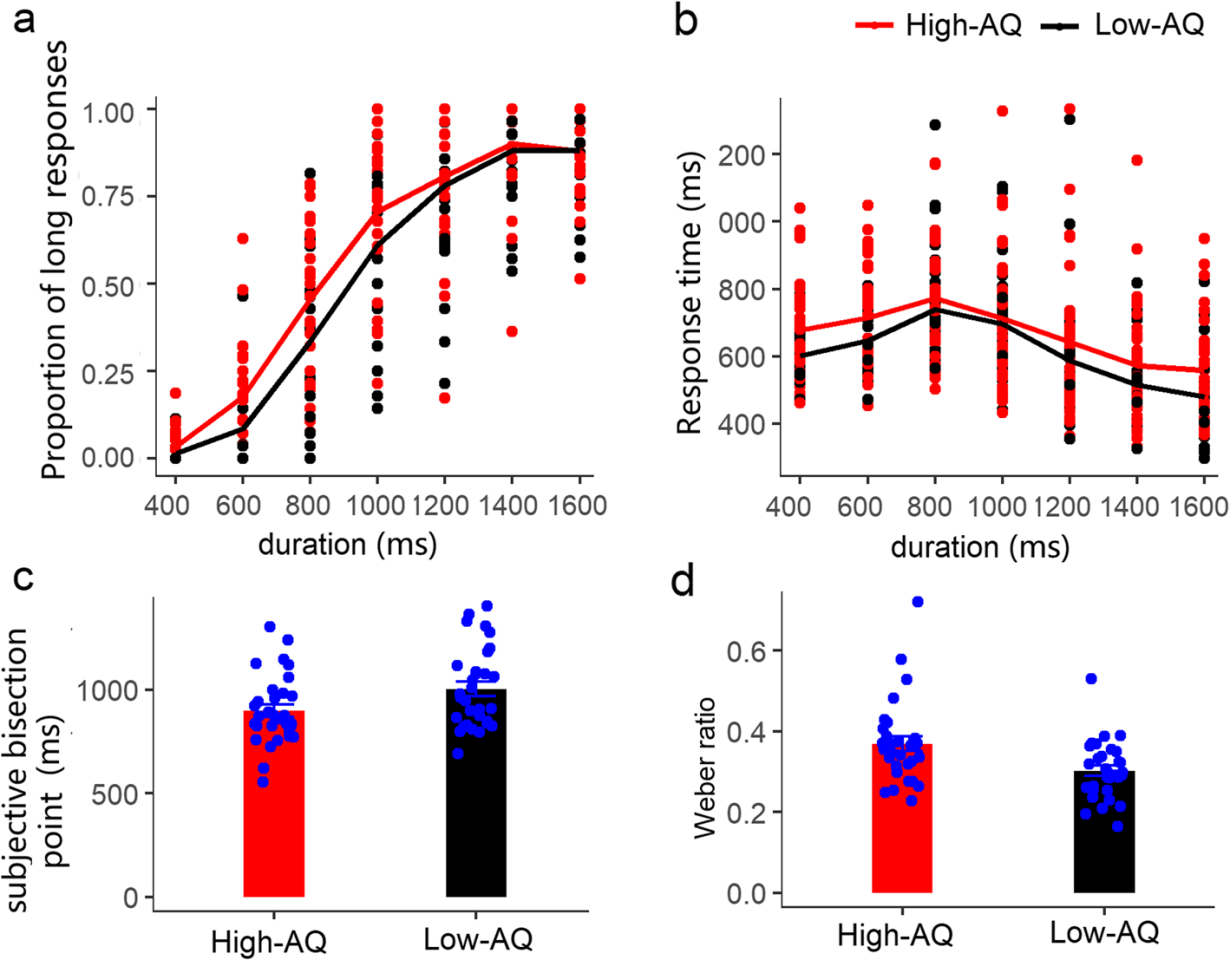
Proportion of the long responses (**a**), response time (**b**), subjective bisection point (**c**), and Weber ratio (**d**) in the temporal bisection task

A two-factor 2 (autistic traits: high-AQ group and low-AQ group) × 7 (duration: 400-1600 ms) repeated-measures analysis of variance (ANOVA) was performed for the proportion of long responses. As shown in Fig. 2a, there was no significant difference in autistic traits, *F*(1, 61) = 2.88, *p* = 0.095, partial *η*^2^ = 0.05. The main effect of duration was significant, *F*(6, 366) = 566.13, *p* < 0.001, partial *η*^2^ = 0.90, and the post-hoc test showed that the difference in the proportion of long responses between 1400 and 1600 ms was not significant, the difference between the other two durations was significant, and the proportion of long responses of the long duration was significantly greater than that for the short duration (*p*s < 0.05). The interaction between duration and autistic traits was significant, *F*(6, 366) = 2.54, *p* = 0.020, partial *η*^2^ = 0.04. Further simple effect analysis showed that the proportion of long responses in the high-AQ group was higher than that in the low AQ group at 400, 600, and 800 ms (*p*s < 0.05), but there was no significant difference in the proportion of long responses between the two groups at the four durations of 1000, 1200, 1400, and 1600 ms (*p*s > 0.05).

For the response time, as shown in Fig. 2b, there was no significant group difference between the high- and low-AQ groups, *F*(1, 61) = 2.55, *p =* 0.116, partial *η*^2^ = 0.04. The main effect of duration was significant, *F*(6,366) = 59.17, *p* < 0.001, partial *η*^2^ = 0.49, where the reaction time at 800 ms was significantly longer than that at other durations; the reaction time at 1600 ms was significantly shorter than that at other durations; and the reaction time at 400 ms was significantly shorter than that at 600, 800, and 1000 ms, and significantly longer than those at 1400 and 1600 ms. The response times at 600 and 1000 ms were significantly greater than those at 1200, 1400, and 1600 ms, and the reaction time at 1200 ms was significantly longer than those at 1400 and 1600 ms (*p*s < 0.05). The interaction between autistic traits and duration was not significant, *F*(6, 366) = 1.11, *p =* 0.357, partial *η*^2^ = 0.02.

The proportion of the long response was fitted with an S-shaped curve to calculate the subjective bisection point (Fig. 2c) and Weber ratio (Fig. 2d). One participant in the high-AQ group’s subjective bisection point was excluded because the value exceeded three times the standard deviation, and one participant in the high-AQ group’s Weber ratio was excluded because his Weber ratio had no real solution. An independent samples *t*-test was conducted to calculate the subjective bisection point and Weber ratio of the two groups. Statistical results showed that the subjective bisection point in the high-AQ group was significantly lower than that in the low-AQ group, *t*(60) = − 2.30, *p =* 0.025. The Weber ratio in the high-AQ group was significantly higher than that in the low-AQ group, *t*(60) = 3.01, *p =* 0.004.

### Discussion

A classical temporal bisection task paradigm was used to explore the difference in time perception between the high-and low-AQ groups in Experiment 1. After learning the standard short duration of 400 ms and the standard long duration of 1600 ms, the participants judged the length of seven durations in the range of 400-1600 ms. We found that the proportion of long responses increased with increasing duration in both the high and low AQ groups, and both groups responded faster to the long and short standard durations, especially for the standard long duration. These results suggest that the high-AQ group can also make a relatively accurate perception within this range of duration. The perception of short durations, ranging from hundreds of milliseconds to several seconds, is the basis of longer temporal processing, so we focused on time perception within this temporal range to explore the influence of autistic traits.

Although there was no difference in the overall trend of time perception between the two groups, a careful analysis of the proportion of long responses showed an interaction between duration and autistic traits. Compared with the low-AQ group, the subjective bisection point was lower, and the proportion of long responses was higher for the high-AQ group at 400, 600, and 800 ms, but there was no significant difference in the longer durations. That is, the high-AQ group provided more conservative responses only for short durations but not for relatively long durations. In addition, we found that the Weber ratio in the high-AQ group was larger, which suggests that their temporal sensitivity was reduced. These results support the idea that temporal processing may be altered in autistic individuals [24, 26], at least within milliseconds to seconds. In the future, it is worth exploring whether the changes are related to the change in the internal clock release rate or the adjustment of attention during timing.

In conclusion, in Experiment 1, we found that individuals with high AQ tended to have perceptual abilities similar to those with low AQ. The difference was that individuals with high AQ tended to overestimate the shorter duration, and the Weber ratio suggested that the temporal sensitivity of individuals with high AQ was relatively decreased, which might be one of the reasons for their overestimation of short durations.

## Experiment 2

### Participants

The participants in Experiment 2 were the same as in Experiment 1.

### Methods

A combination of an identity-association learning task and temporal bisection task was used in Experiment 2. The participants were required to complete an identity association learning task before completing the temporal bisection task. Referring to the self-shape association paradigm [33], the task was divided into three stages. In the first stage, the participant was asked to imagine the square, triangle, and circle as the self, a friend of the same sex, and a stranger, respectively. The color and angle of the outer circle of the shapes were the same for the three geometric shapes. The second stage was an identity association exercise with a total of 18 trials. A geometric shape was randomly presented in the center of the screen, and three identity labels “me,” “friend,” and “stranger” were displayed below the shape. The participants selected the identity corresponding to the current geometric shape using “B,” “N,” and “M” on the keyboard. Feedback was provided based on the responses of the participants at this stage. The third stage was a formal experiment on identity association, which proceeded similarly to the second stage, except that no feedback was provided. There were 216 trials in the formal identity association stage, and each shape was randomly presented 72 times. The association between shape and identity information was balanced among participants. After identity association learning, the participants completed the temporal bisection task immediately. This part was the same as in Experiment 1, except that the gray diamond was replaced with three types of geometric shapes representing the self, a friend of the same sex, and a stranger. There were 588 trials in the temporal bisection task, and each of the seven durations appeared 28 times. The interval of each trial was random within 1–3 s.

### Results

Data from three participants were excluded because their accuracy exceeded by three times the standard deviation, and the remaining 60 participants (30 in the high-AQ group and 30 in the low-AQ group) were included in the statistical analysis of accuracy and response time in the identity-association learning task and the proportion of long response and response time in the temporal bisection task.

For the identity-association learning task, a 2 (autistic traits: high-AQ group and low AQ group) × 3 (identity: self, friend, and stranger) repeated-measures ANOVA was used for the accuracy and response time. The accuracy of the two groups was greater than 97.05%, and no significant difference was found between the two groups. The main effect of identity was significant, *F*(2, 116) = 4.40, *p =* 0.014, partial *η*^*2*^ = 0.07, and the post-hoc test showed that accuracy for friends was significantly higher than that for strangers (*p =* 0.004). The results of the response time showed that there was no significant difference between the two groups, *F*(1, 58) = 2.13, *p* = 0.15, partial *η*^*2*^ = 0.04. The main effect of identity was insignificant: *F*(2, 116) = 2.11, *p =* 0.125, and partial *η*^*2*^ = 0.04.

**Fig. 3:**
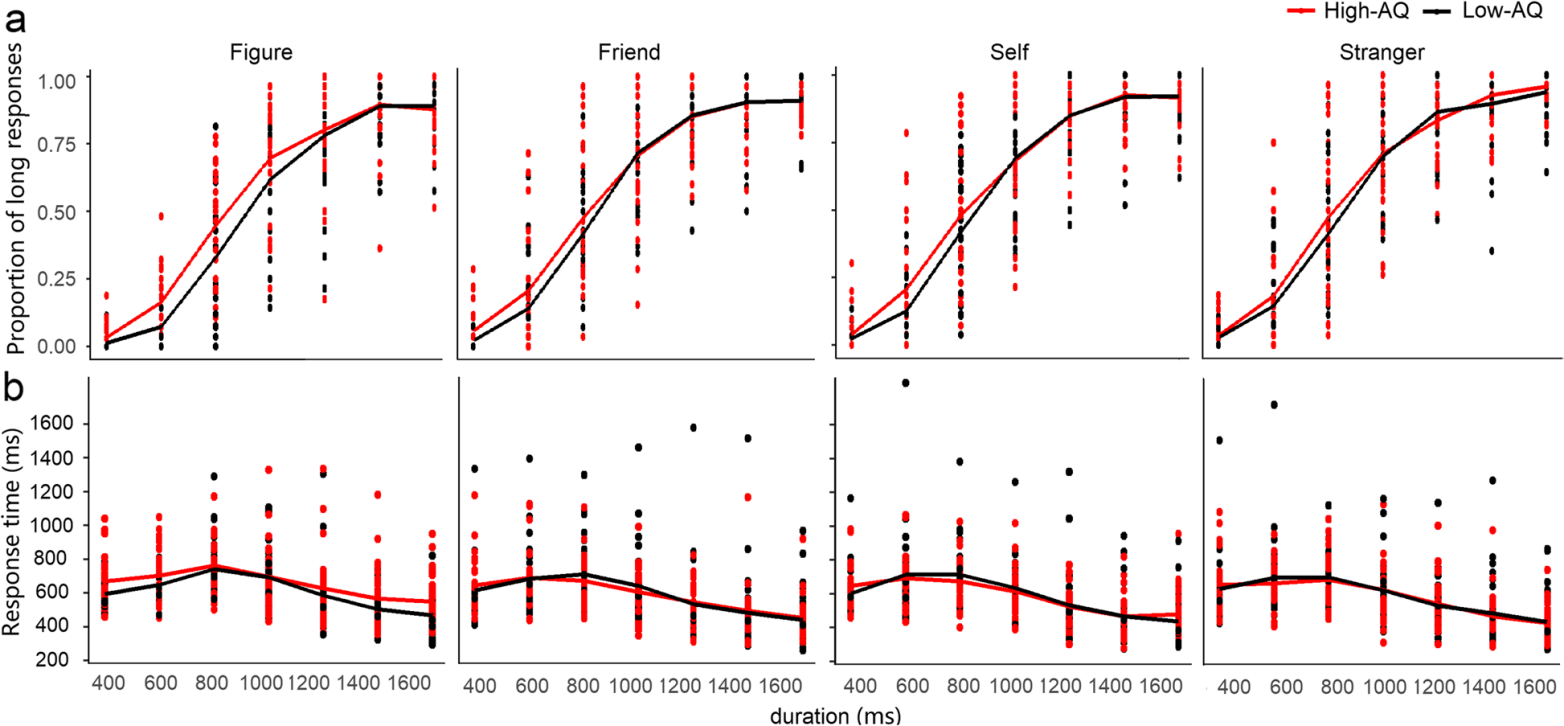
Proportion of long responses (**a**) and response time (**b**) in a combination of temporal bisection task and identity-association task

Regarding the temporal bisection task involving identity information, two participants (one in the high-AQ group and one in the low-AQ group) were excluded because the fitting parameters (*R*^2^) exceeded three times the standard deviation. Therefore, the data of the remaining 58 participants (29 in the high-AQ group and 29 in the low-AQ group) were entered into the statistical analysis of the proportion of long responses, subjective bisection points, and Weber ratios. Combined with the condition of no identity information, the proportion of long responses was performed using a three-factor repeated-measures ANOVA of 2 (autistic traits) × 7 (duration) × 4 (identity), the results of which are shown in Fig. 3a. There was no significant difference between the high and low AQ groups, *F*(1, 56) = 0.91, *p* = 0.345, partial *η*^*2*^ = 0.02. The main effect of identity was significant, *F*(3, 168) = 5.48, *p* = 0.001, partial *η*^*2*^ = 0.09. The post-hoc test showed that the proportion of long responses for no identity was significantly lower than for self (*p* = 0.022), friends (*p* = 0.022), and strangers (*p* = 0.014). The main effect of duration was significant, *F*(6, 336) = 806.10, *p* < 0.001, partial *η*^*2*^ = 0.094, and a post-hoc test showed that the proportion of long responses was significantly different between each pair of long and short durations (*p*s < 0.001), except for the duration pair of 1600 and 1400 ms (*p* = 0.923). All second -and third-order interactions were not statistically significant (*p*s > 0.05).

Here we are specifically interested in autistic traits related changes, and the curves showed a different trend in high-AQ and low-AQ group, although the interactions between autistic traits and other variables was insignificant statistically. We further performed a two-factor repeated-measures ANOVA of 7 (duration) × 4 (identity) in the high-AQ and low AQ-groups, respectively for the proportion of long response. A significant difference in identity only appeared in the low-AQ group, *F*(3, 84) = 5.80, *p* = 0.001, partial *η*^*2*^ = 0.17. The post-hoc test showed that the proportion of long responses for no identity was significantly lower than for self (*p* = 0.022), friends (*p* = 0.017), and strangers (*p* = 0.011). The main effect of duration was significant for the low AQ-group, *F*(6, 168) = 536.21, *p* < 0.001, partial *η*^*2*^ = 0,095; and a post-hoc test showed that the proportion of long responses was significantly different between each pair of long and short durations (*p*s ≤ 0.001), except for the duration pair of 1400 and 1600 ms (*p* = 0.230). In the high-AQ group, the main effect of identity was insignificant, *F*(3, 84) = 1.10, *p* = 0.352, partial *η*^*2*^ = 0.04. The main effect of duration was significant, *F*(6, 168) = 316.52, *p* < 0.001, partial *η*^*2*^ = 0.92, and a post-hoc test showed that the proportion of long responses was significantly different between each pair of long and short durations (*p*s ≤ 0.001), except for the duration pair of 1400 and 1600 ms (*p* = 0.799).

A three-factor 2 (autistic traits) × 7 (duration) × 4 (identity) repeated-measures ANOVA was performed on the response time of the temporal bisection task. As shown in Fig. 3b, the results showed that there was no significant difference between the high- and low-AQ groups, *F*(1, 56) = 0.10, *p* = 0.751, partial *η*^*2*^ < 0.01. The main effect of identity was significant, *F*(3, 168) = 4.69, *p* = 0.004, partial *η*^*2*^ = 0.08, and a post-hoc test showed that the response time of no identity was significantly longer than that of self (*p* = 0.028) and stranger (*p* = 0.019). The main effect of duration was significant, *F*(6, 336) = 139.11, *p* < 0.001, partial *η*^*2*^ = 0.71; a further post-hoc test showed that the RTs for the durations of 600 and 800 ms were longer than those on other durations (ps ≤ 0.002), and the RTs for the durations of 400 and 1000 ms were longer than that for 1200 ms, 1400 ms, and 1600 ms (*p*s < 0.001). The interaction between identity and duration was significant, *F*(18, 1008) = 4.33, *p <* 0.001, partial *η*^*2*^ = 0.07; there was no significant difference among all identities at short durations of 400 ms (*p*s ≥ 0.116). However, for long durations, including 800, 1000, 1200 ms, the response time of no identity was significantly longer than that of friends, self, and strangers (*p*s ≤ 0.026). At the duration of 600 ms, the response time of the self was significantly longer than that of the stranger; at the durations of 1400 ms, the response time of no identity was significantly longer than that of the self and stranger; at the durations of 1600 ms, the response time of no identity was significantly longer than that of the self and friend, and the response times of self and friend were significantly longer than that of the stranger. Other second-order and third-order interactions were not statistically significant (*p*s > 0.05).

**Fig. 4:**
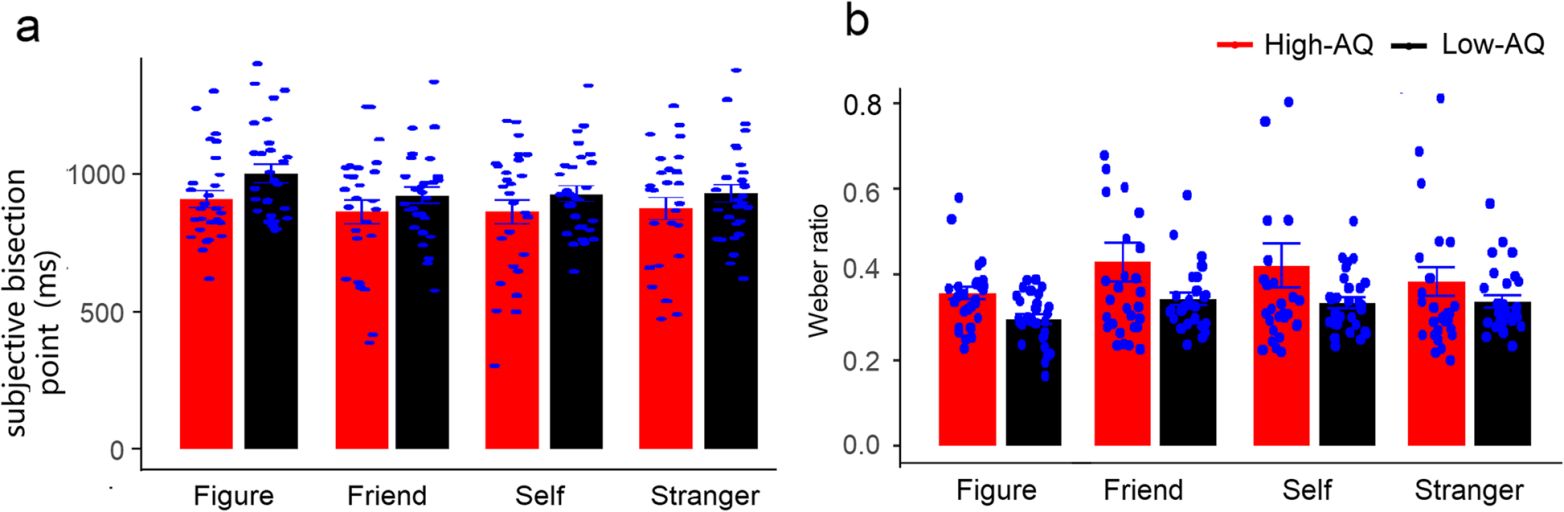
Weber ratio (**a**) and subjective bisection point (**b**) in a combination of temporal bisection task and identity-association task

A two-factor 2 (autistic traits) × 4 (identity) repeated-measures ANOVA was performed for the subjective bisection point and Weber ratios, as shown in Fig. 4. One high-AQ group participant ‘s subjective bisection point was excluded because the value exceeded three times the standard deviation, and the Weber ratio of two high-AQ group participants was excluded because the value exceeded three times the standard deviation. The results of the subjective bisection points showed that the main effect of identity was significant, *F*(3, 165) = 4.89, *p* = 0.003, partial *η*^*2*^ = 0.08, and the subjective bisection of no identity was significantly higher than that of friends (*p* = 0.024), self (*p* = 0.023), and strangers (*p* = 0.038). There were no significant group differences or interactions between autistic traits and identity (*p* > 0.05).

The results of the Weber ratio showed that the main effect of identity was significant, *F*(3, 162) = 3.88, *p =* 0.010, partial *η*^*2*^ = 0.07, and the Weber ratio of friends was significantly higher than that of no identity (*p* = 0.014). There was no significant difference between the high- and low-AQ groups in the Weber ratio, *F*(1, 54) = 1.89, *p* = 0.175, partial *η*^*2*^ = 0.03. The interaction between autistic traits and identity was not significant, *F*(3, 162) = 1.08, *p* = 0.360, partial *η*^*2*^ = 0.02.

### Discussion

In Experiment 2, we combined an identity-association learning task and temporal bisection task to investigate the relationship between subthreshold autistic traits and time perception under different identities. In the identity-association learning stage, both groups showed high accuracy, and no significant group difference was found for the response time and accuracy, suggesting that both groups could establish an association between geometric shapes and identity information. This is the basis for the next time perception task, that is, the different performances on the temporal bisection task are not caused by different performances on the identify-association task. However, this does not mean that the sensitivity to identity and level of involvement in identity information are the same between the two groups.

Once the association between identity information and geometric shapes was established, we observed changes in almost every indicator with the involvement of identity information. In the condition of no identity, the proportion of long responses was significantly lower than in the three identity conditions, the subjective bisection point was significantly higher than that for self and stranger conditions, and the Weber proportion was significantly lower than that of other identity information. Based on a temporal range from 400 to 1600 ms, 1000 ms should be the objective bisection point. The results of the proportion of long responses (all are > 0.5) and subjective bisection points (all are < 1000 ms) suggested that participants tended to overestimate duration within the range of 400-1600 ms. However, participants further overestimated the duration more with than without identity involvement. Combined with the results of the higher Weber ratio, this may suggest that the overestimation may be related to the decrease in time sensitivity.

In addition, we found for the proportion of long responses, the difference between no identity and identity seemed more obvious in the low-AQ group. This is consistent with the previous hypothesis that the low-AQ group is more sensitive to interpersonal information and therefore shows more susceptibility to time perception. In contrast, the high-AQ group seemed to show a relatively stable time perception of the involvement of interpersonal information. It is worth noting that further evidence is still needed to test it although the inference sounds interesting.

## Total discussion

Temporal processing forms the basis of almost all human cognitive processes. In previous studies, clinical populations with high autistic traits were reported to have changes in temporal processing, but their performance within different temporal ranges is controversial [26, 38]. Given the fact that long-duration processing may be more complicated and may be involved in more cognitive processing [27], in the current study, we focused on time perception in non-clinical individuals with high autistic traits within a temporal range from 400 to 1600 ms. We explored perceptual accuracy, the direction of deviation, and temporal sensitivity using the proportion of long response, subjective bisection point, and the Weber ratio, respectively, in a temporal bisection task. The results show that the overall trend and response pattern are similar for the high-and low-AQ groups within the range of hundreds of milliseconds to seconds. Both groups had an increased proportion of long responses along with an increase in duration length, indicating that they had a rather accurate time perception.

More detailed analysis indicated that individuals with high AQ tended to overestimate shorter durations of this range, and the trend of overestimation disappeared at the longer end of the temporal range but did not show an underestimation of long durations. An interesting phenomenon in time processing is that individuals often show a “central tendency” that approaches the average, that is, a tendency to underestimate a relatively long period and overestimate a relatively short one. This “central tendency” is a flexible cognitive strategy to improve accuracy at the expense of sensitivity when an individual’s perceptual ability is insufficient to make a clear judgment. Previous studies have found that children with ASD have a higher “central tendency” than typically developing children of the same age, although their central tendency was much less than predicted by theoretical modeling [25]. Here, we found that the high-AQ group performed more conservative responses only for short durations, which is not exactly consistent with the “central tendency.” We only observed a trend in which the overestimation gradually decreased from relatively short to relatively long durations. The longest duration (1600 ms) of this range was relatively short compared with the larger time ranges. Thus, it may be necessary to extend the duration to detect changes at the other end of the duration range in the future. In addition, individuals with high-AQ had a higher Weber ratio than individuals with low AQ in the first experiment without identity-association learning, which suggests that they have decreased temporal sensitivity. This could be one reason why individuals with a high AQ tend to overestimate shorter durations.

Previous studies have shown that interpersonal information is specific to human beings, such as name, face, eye, and even voice, which can mark identity [39, 40]. Individuals with high autistic traits show an atypical pattern for processing interpersonal information [11, 41, 42], which may further affect basic cognitive processing, including perception, attention and memory [28]. Previous studies have shown that stimuli with subjective salience can affect duration estimation; participants overestimated the duration of their names [43]. In our study, to exclude the influence of different physical attributes from face or name, the identity association learning task was combined with the classical temporal bisection task [33, 34], and participants were required to complete association learning of identity and shapes before the time perception task. The results showed that the involvement of identity led to an overestimation of duration and decreased temporal sensitivity. One possible reason for these results is that introducing identity information increases the arousal level of individuals. Sui et al. (2012) indicated that associating a stimulus with the self modulates its subsequent perceptual processing, which may operate by identity-associated shapes that automatically evoke the reward system [33]. According to recent definitions, emotions can be conceptualized as psychophysiological states that reflect an organism’s appraisal of the meaning, relevance, and value of events in the world. Tacikowski and Nowicka (2010) suggest that self-name and self-face could be regarded as emotional stimuli [44]. Based on the dominant model of time perception, our ability to perceive time relies on a pacemaker-accumulator clock; arousal is thought to increase the speed of the pacemaker, resulting in a longer perceived duration, reducing subjective bisection points, and increasing the proportion of long responses [45–47]. In addition, our results indicate that response time is reduced when identity information is involved, which indirectly supports the speculation of increased arousal. This speculation needs to be explored further in the future.

We also observed a trend that the effect of interpersonal information on the proportion of long responses seemed more obvious in the low-AQ group rather than in the high-AQ group. If future studies can supply more robust evidence, this may implies that individuals with high AQ are less sensitive to interpersonal information, which may becomes a protective factor of cognitive processing, showing the characteristic of being more stable and less easily disturbed by interference. In addition, the temporal processing is limited to the range of perception in this study. In future studies, if we enlarge the temporal range, more factors may be introduced into the long-duration processing, and changes in long-duration perception may be an accumulation, amplification, and adjustment of short-duration perception.

## Limitations

The current study has some limitations. First, the AQ, a self-reported tool of autistic traits, was used to group high and low levels of autistic traits. Although the AQ has been widely used [2, 48, 49], and the revised Chinese version has relatively high reliability and validity [36], it would be more valuable if a combination of self-evaluated and other-evaluated questionnaires was used in future studies. The participants selected in this study were distributed at the high and low ends of autistic traits; thus, the correlation between autistic traits and sensitivity or deviation of time perception was not explored in detail. Second, the study used an association-learning task to establish associations between identities and shapes. Although the design can avoid physical attribute differences of several identities, and participants showed relatively high accuracy for the identity-shapes association learning, the involvement of identity information is dependent on association strength and speed. Third, more robust evidence was needed to prove the trend that the effect of interpersonal information on the proportion of long responses seemed more obvious in the low-AQ group than in the high-AQ group. In addition, this study used a temporal bisection task in the range of 400-1600 ms, but the influence of autistic traits and interpersonal information on time processing needs to be further explored within different temporal ranges using different timing paradigms.

## Conclusion

In this study, we used a temporal bisection task combined with identity-association learning to explore the effect of autistic traits on time perception in the range of 400-1600 ms and investigated the effect of the involvement of different identities through social identity associative learning. The results showed that individuals with high AQ tended to overestimate the shorter duration of this range and have lower subjective bisection points and higher Weber ratios than those with low AQ, suggesting that their temporal sensitivity decreased. With the involvement of identity information, the proportion of long responses for no identity was significantly lower than for the three identities. The Weber ratio of no identity was lower than that of other identities.

## Data Availability

The datasets used and/or analyzed during the current study are available from the corresponding author upon reasonable request.

## Abbreviations

ASD: Autism Spectrum Disorder
AQ: Autism Spectrum Quotient
High AQ: High levels of autistic traits
Low AQ: Low levels of autistic traits
